# Supplementary material for: COZOID: contact zone identifier for visual analysis of protein-protein interactions
Source: BMC Bioinformatics. 2018 Apr 6;19:125. doi: 10.1186/s12859-018-2113-6 (PMC5889581; doi:10.1186/s12859-018-2113-6)
Supplement: Supplementary file 2 — Software build. Executable binary file of the software tool. (ZIP 118,784 kb) [file 12859_2018_2113_MOESM2_ESM.zip › cozoid/platform/docs/testng-6.5.1-javadoc.zip!/org/testng/IMethodInterceptor.html]

IMethodInterceptor (TestNG)


---


|  |  |  |
| --- | --- | --- |
| **Overview** \| **Package** \| **Class** \| **Tree** \| **Deprecated** \| **Index** \| **Help** | |  |
| **PREV CLASS**   **NEXT CLASS** | **FRAMES**    **NO FRAMES**     **All Classes** |  |
| SUMMARY: NESTED \| FIELD \| CONSTR \| METHOD | DETAIL: FIELD \| CONSTR \| METHOD |  |


---


## org.testng Interface IMethodInterceptor

**All Superinterfaces:**: ITestNGListener

---

``` public interface IMethodInterceptor extends ITestNGListener ```

This class is used to alter the list of test methods that TestNG is about to run.

An instance of this class will be invoked right before TestNG starts invoking test methods.
Only methods that have no dependents and that don't depend on any other test methods will
be passed in parameter. Implementers of this interface need to return a list of `IMethodInstance`
that represents the list of test methods they want run. TestNG will run these methods in the
same order found in the returned value.

Typically, the returned list will be just the methods passed in parameter but sorted
differently, but it can actually have any size (it can be empty, it can be of the
same size as the original list or it can contain more methods).

The `ITestContext` is passed in the intercept method so that implementers can set user values
(using `IAttributes.setAttribute(String, Object)`), which they can then look up
later while generating the reports.

**Author:**
:   cbeust

---

| **Method Summary** | |
| --- | --- |
| `java.util.List<IMethodInstance>` | `intercept(java.util.List<IMethodInstance> methods, ITestContext context)` |

**Method Detail**

### intercept

```
java.util.List<IMethodInstance> intercept(java.util.List<IMethodInstance> methods,
                                          ITestContext context)
```


---


|  |  |  |
| --- | --- | --- |
| **Overview** \| **Package** \| **Class** \| **Tree** \| **Deprecated** \| **Index** \| **Help** | |  |
| **PREV CLASS**   **NEXT CLASS** | **FRAMES**    **NO FRAMES**     **All Classes** |  |
| SUMMARY: NESTED \| FIELD \| CONSTR \| METHOD | DETAIL: FIELD \| CONSTR \| METHOD |  |


---
